# Chemokines in the balance: maintenance of homeostasis and protection at CNS barriers

**DOI:** 10.3389/fncel.2014.00154

**Published:** 2014-05-28

**Authors:** Jessica L. Williams, David W. Holman, Robyn S. Klein

**Affiliations:** ^1^Department of Internal Medicine, Washington University School of MedicineSt. Louis, MO, USA; ^2^Infectious Diseases Division, Decision Resources GroupBurlington, MA, USA; ^3^Department of Pathology and Immunology, Washington University School of MedicineSt. Louis, MO, USA; ^4^Department of Anatomy and Neurobiology, Washington University School of MedicineSt. Louis, MO, USA

**Keywords:** chemokines, central nervous system, blood brain barrier, homeostasis, vasculature, choroid plexus, meninges, neurogenesis

## Abstract

In the adult central nervous system (CNS), chemokines and their receptors are involved in developmental, physiological and pathological processes. Although most lines of investigation focus on their ability to induce the migration of cells, recent studies indicate that chemokines also promote cellular interactions and activate signaling pathways that maintain CNS homeostatic functions. Many homeostatic chemokines are expressed on the vasculature of the blood brain barrier (BBB) including CXCL12, CCL19, CCL20, and CCL21. While endothelial cell expression of these chemokines is known to regulate the entry of leukocytes into the CNS during immunosurveillance, new data indicate that CXCL12 is also involved in diverse cellular activities including adult neurogenesis and neuronal survival, having an opposing role to the homeostatic chemokine, CXCL14, which appears to regulate synaptic inputs to neural precursors. Neuronal expression of CX_3_CL1, yet another homeostatic chemokine that promotes neuronal survival and communication with microglia, is partly regulated by CXCL12. Regulation of CXCL12 is unique in that it may regulate its own expression levels via binding to its scavenger receptor CXCR7/ACKR3. In this review, we explore the diverse roles of these and other homeostatic chemokines expressed within the CNS, including the possible implications of their dysfunction as a cause of neurologic disease.

## Introduction

Our understanding of the role of chemokine expression in the adult central nervous system (CNS) has shifted away from viewing these molecules primarily as proinflammatory mediators and more towards their ability to exert neuroprotective and reparative functions. This is especially the case for chemokines categorized as “homeostatic”, based on their constitutive expression in thymic and lymphoid tissues (CCL14, CCL19, CCL20, CCL21, CCL25, CCL27, CXCL12 and CXCL13), where they regulate the migration of leukocytes during immune surveillance (Bachmann et al., 2006; Ito et al., 2011). Of these chemokines, CCL19, CCL20, CCL21, CCL27 and CXCL12 are expressed within uninflamed CNS tissues (van der Meer et al., 2000; Stumm et al., 2002), particularly at CNS endothelial barriers (Kivisäkk et al., 2004; Reboldi et al., 2009). Two additional chemokines, CX_3_CL1 and CXCL14, are also expressed at high levels in the normal CNS, primarily by neurons (Harrison et al., 1998; Huising et al., 2004; Banisadr et al., 2011). Ligands as well as receptors for several CNS homeostatic chemokines are expressed by neural stem cells (Huising et al., 2004; Kokovay et al., 2010), while others can be found on microglia and neurons (Sheridan and Murphy, 2013). These chemokines and their receptors are therefore involved in a range of homeostatic processes including immune surveillance, neuro/gliogenesis and modulation of synaptic transmission. This review will discuss how homeostatic chemokines protect and maintain normal CNS functions.

## Chemokines Regulate CNS Immune Privilege and Surveillance

### CNS Barriers

**Figure 1:**
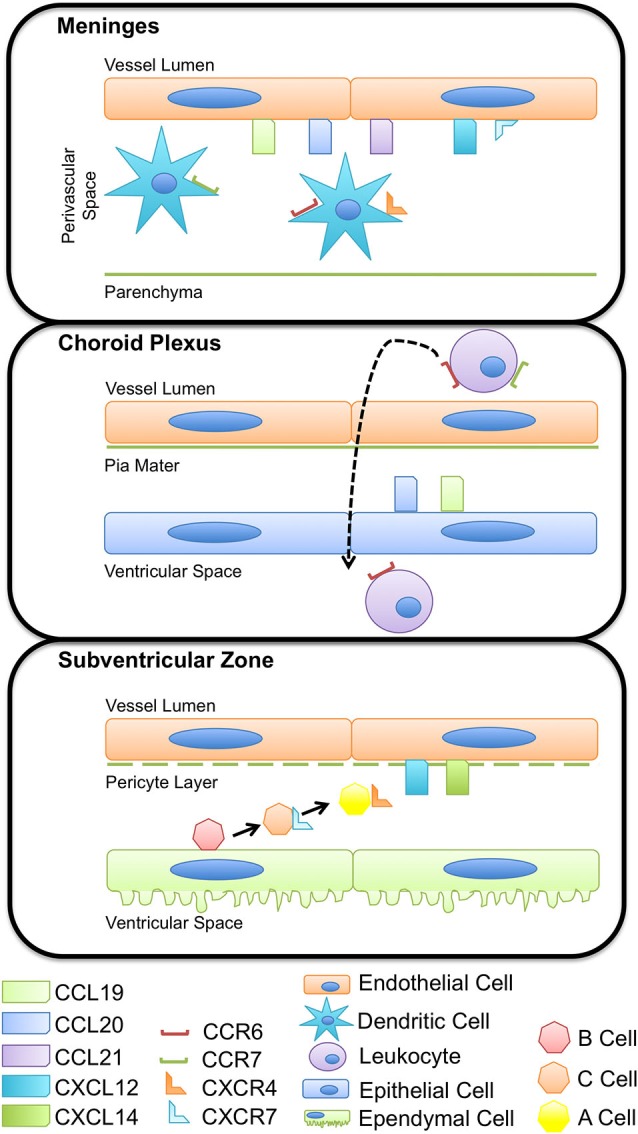
**Chemokines regulate immune and progenitor cell homeostasis at multiple CNS barriers**. Chemokines expressed by the CNS vasculature have a role in regulating immune cell and neural progenitor cell occupancy within perivascular spaces. Expression of CCL19, CCL20, and CCL21 on meningeal vessels maintain dendritic cell populations important for immunosurveillance within the boarders of the glia limitans, limiting antigen presentation cell access to the CNS parenchyma. The vasculature of the choroid plexus expresses a variety of adhesion molecules and chemokines, including CCL19 and CCL20 that influence immune cell adhesion, rolling, and extravasation across the endothelium and pia mater. CCR6^+^ and CCR7^+^ leukocytes enter CSF-containing ventricles and circulate through the CNS, surveying for antigen and other cues indicative of inflammation. Subventricular zone (SVZ) neural precursors receive external cues, namely CXCL12, from the vasculature, that prompts proliferation, differentiation, and migration. Ependymal cells form a thin epithelial-like lining of the ventricular system and enclose the niche in which SVZ progenitors thrive.

Homeostasis of the CNS is maintained within strict limits by anatomical and immunological barriers that restrict access of pathogens, solutes, and to an extent, immune cells, to the brain parenchyma. This review focuses on two of these barriers in particular; the blood–cerebrospinal fluid (CSF) barrier and blood brain barrier (BBB), which prevent the exchange of cells and solutes between the blood and CSF or brain parenchyma, respectively. The BBB is comprised of specialized endothelial cells of the cerebral microvasculature, surrounding pericytes, and astrocytic endfeet, while the blood–CSF barrier is largely made up of the fenestrated endothelium of the choroid plexus. In addition to these anatomical barriers, the expression of chemokines and chemokine receptors at the BBB and blood-CSF barrier serves as an immunological checkpoint and prevents (during non-inflammatory/homeostatic conditions) or promotes (during neuroinflammation) the infiltration of circulating leukocytes into the deeper CNS parenchyma and ventricular or subarachnoid CSF spaces (Figure 1).

As in peripheral lymphoid tissues, expression of chemokines and their cognate receptors within the CNS are highly regionalized and regulated in a tissue-dependent manner (Réaux-Le Goazigo et al., 2013). In particular, endothelial cell chemokines expressed at the BBB can translocate from the abluminal to luminal surfaces of post-capillary venules, thereby exerting effects on circulating leukocytes. Thus, a critical aspect of chemokine function at the BBB, as in peripheral tissues, is their localization along endothelial cell surfaces and binding to extracellular matrix proteins and glycosaminoglycans (GAGs). The discovery of chemokines’ ability to direct leukocyte migration was largely informed through *in vitro* studies of the movement of leukocytes towards increasing concentrations of solubilized chemokines (Zachariae, 1993). These analyses perhaps inappropriately fostered the notion that within tissues, leukocytes similarly respond to soluble chemokine gradients; however it is now recognized that the extracellular matrix and GAGs localize and concentrate chemokines, preventing their rapid diffusion and loss of chemotactic effects (Hamel et al., 2009). In particular, chemokines have been shown to bind with high affinity to heparan sulfate chains of heparan sulfate proteoglycans, immobilizing chemokines and leading to the formation of chemokine gradients on endothelial surfaces (Johnson et al., 2005; Parish, 2006).

The concept of immune privilege was originally conceived as a result of experiments that found antigenic material, including foreign tumors and tissue grafts, failed to elicit a systemic, T cell-mediated immune response when implanted into the CNS parenchyma (reviewed in Galea et al., 2007). While the term immune privilege implies an absence of immunological response within the CNS, it is now recognized that CNS immune privilege is not absolute but rather very elaborately controlled. Several cellular and molecular components that comprise the CNS barriers are responsible for limiting the immune response under homeostatic conditions.

Microglia and perivascular macrophages are critical components of CNS immune surveillance and protection. While microglia share morphology and many functions with perivascular macrophages, their ontogeny differs. Microglia are myeloid phagocytes (Nimmerjahn et al., 2005) derived from the yolk sac during early development (Ginhoux et al., 2010; Kierdorf et al., 2013) and are found throughout the CNS of adults. Once activated, microglia initiate a classical innate immune response, similar to that elicited by peripheral macrophages, and facilitate activation of adaptive immunity, secreting inflammatory cytokines and presenting antigen(s) to reactive lymphocytes. In adults, perivascular macrophages originate from stem cell niches in the bone marrow and localize to perivascular spaces, confined by the BBB around blood vessels, via the circulation (Neumann and Wekerle, 2013). Perivascular macrophages function similar to peripheral macrophages, and thus they are crucial for antigen presentation to and reactivation of lymphocytes, making them a critical component of CNS-defense against invading pathogens. Further, elimination of these perivascular cells enhances responses to inflammatory stimuli, including LPS, suggesting that perivascular macrophages may have a role in controlling initial host-pathogen responses within the CNS (Serrats et al., 2010). While a repertoire of chemokine and chemokine receptors are expressed by microglia and perivascular macrophages, their function with regard to antigen presentation is not well understood. However, it is likely that chemokines underlie the localization of these cells within their respective CNS compartments. Together, microglia and perivascular macrophages form another layer of CNS protection, comprising a cellular barrier to facilitate protection against invading pathogens as well as immune-mediated bystander CNS injury.

### The Choroid Plexus and Meningeal Barriers

The choroid plexus and the meninges represent pivotal modified cellular barriers between the blood and CSF or parenchymal compartments, respectively. The choroid plexus is largely considered a circumventricular organ, localized in the ventricles, and constitutes one of the interfaces between the blood and the CSF (Strazielle and Ghersi-Egea, 2000; Schulz and Engelhardt, 2005). The epithelial cells of the choroid plexus secrete CSF and thus largely contribute to brain homeostasis, adjusting intracranial volume, buffering extracellular solutes, and supplying cells of the CNS with micronutrients (Strazielle and Ghersi-Egea, 2000). The vascular endothelial cells of the choroid plexus are unique from those of the BBB as they lack tight junctions, more readily enabling diapedesis of cells. Further, as opposed to postcapillary venules within the parenchyma, which require cells to traffic across two basement membranes, meningeal capillaries have only a one-layer structure (Wilson et al., 2010). In addition to representing points of access for circulating immune cells, the choroid plexus and the meninges also host a local population of antigen presenting cells. In rats and humans, choroid plexus- and meninges-associated dendritic cells (DCs) have been identified, which express major histocompatibility class II (MHC II) and present antigen to circulating lymphocytes (McMenamin, 1999). These DCs are known to sample the environment by extending processes between adjacent choroid plexus epithelial cells (Serot et al., 2000), making the choroid plexus and the meninges major sites of immunosurveillance (Figure 1).

**Figure 2:**
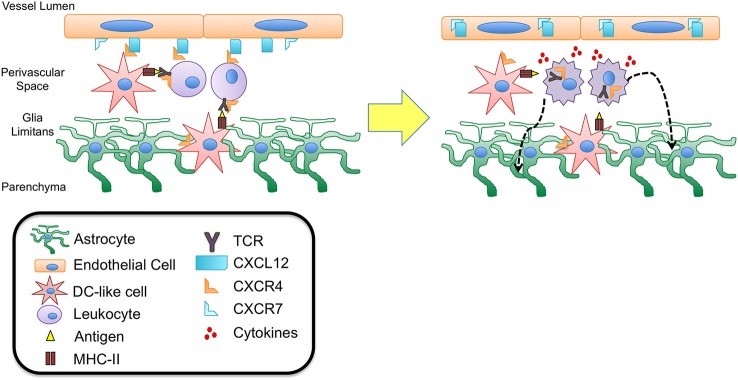
**CXCL12 fate determination of perivascular T cells**. DC-like cells have been identified in the perivascular space as well as in the juxtavascular parenchyma abutting astrocytic end feet and extending processes into the basement membrane of the glia limitans. These cells express CD11c but lack MHC II expression suggesting they may be quiescent DCs capable of recognizing antigen during routine immune surveillance. It is possible that upon antigen uptake these cells are activated to up-regulate MHC II expression as well chemokine receptors such as CXCR4. Interaction with CXCL12 expressed at the abluminal side of the BBB endothelium could thus function to retain these cells as well as circulating lymphocytes within the perivascular spaces, facilitating antigen presentation and recognition. Upon binding to antigen presented in MHC II complexes, the T cell antigen receptor (TCR) expressed on T cells heterodimerizes with CXCR4, initiating intracellular signaling cascades that lead to T cell activation and migration. Recent studies have demonstrated that TCR-activated β-arrestin signaling down-regulates T cell surface CXCR4 expression, perhaps removing an important retention cue within the perivascular niche, thereby promoting entry of T cells into deeper parenchymal tissue. This process may also be promoted by the release of cytokines due to TCR binding and T cell activation. Inflammatory cytokines have been shown to up-regulate levels of CXCL12 as well as the scavenger chemokine receptor CXCR7. CXCR7 is capable of binding to and internalizing CXCL12, leading to loss of polarity at the BBB. Relocation of CXCL12 from abluminal to luminal endothelial surfaces at the BBB has been shown to lead to extensive leukocyte infiltration and neuroinflammation in experimental autoimmune encephalomyelitis (EAE) and multiple sclerosis (MS).

The localization of DCs in close proximity to the vessels of the choroid plexus and meninges suggests that these cells express a set of chemokines that limit their mobility out of these compartments. Further, DC turnover dictates that these cells exhibit temporal expression of chemokine localizing cues to facilitate their egress from the circulation and into the choroid plexus and meningeal compartments (Chinnery et al., 2010). DCs are known to express several chemokine receptors including CXCR3, CCR6, CCR7, and CXCR4 as well as some others, depending on their stage of maturation (Charles et al., 2010), and numerous chemokines have been identified in recruiting DCs into the brain parenchyma during neuroinflammation as well as prion disease, viral encephalitis, brain ischemia, parasitic and bacterial CNS infections (Clarkson et al., 2012). However, it remains unclear which chemokines are involved in maintaining DCs within CNS compartments during immune surveillance. Under homeostatic conditions, the presence of DCs in the stroma of the chroroid plexus as well as meningeal blood vessels suggest a role for chemokines that are constitutively expressed at these locations, such as CCL19, CCL20 and CCL21 (Figure 2). Recent studies have also demonstrated vessel-associated, DC-like cells in the CNS perivascular space that extend cell processes into the basement membrane of the glia limitans in the absence of inflammation. These DC-like cells expressed CD11c, but not MHC II (Prodinger et al., 2011). The authors of this study speculate that these cells may represent a subpopulation of microglia, cells of the monocytic lineage, or immature or quiescent DCs capable of recognizing and presenting antigen. Since these cells extend processes into the glia limitans, it is possible that they are capable of sampling and presenting antigen within the perivascular space. It is possible that abluminally expressed CXCL12, which binds to CXCR4 expressed by DCs, at the BBB may be important in retaining these cells within close proximity to the microvasculature, allowing interactions with surveying T cells, but preventing access to deeper parenchymal tissue.

Given that mature DCs up-regulate expression of cognate chemokine receptors, including both CCR7 and CXCR4 (Sallusto et al., 1998), it is possible that expression of these receptors localize mature DCs to the chroroid plexus (via CCL21) and BBB (via CCL19 and CXCL12), thus facilitating interactions between circulating lymphocytes and these antigen presenting cells. Antigen presented by mature DCs and recognized by T cell receptors (TCRs) results in heterodimerization between TCRs and CXCR4 and is necessary to initiate activation, cytokine secretion, and T cell migration. Further, CXCL12 has been shown to enhance T cell responses via costimulation of the TCR (Smith et al., 2013), suggesting that CXCL12 expressed in the perivascular niche may play an important role in mediating TCR activation during antigen presentation. Furthermore, heterodimerization of CXCR4 and TCR (Kremer et al., 2011) triggers TCR signaling via β-arrestin-1 that results in down-regulation of CXCR4 (Schneider et al., 2009; Fernández-Arenas et al., 2014), perhaps limiting CXCL12 action at the BBB and preventing prolonged retention of T cells within the perivascular space (Figures 1 and 2).

The chemokine CXCL12, also known as stromal cell-derived factor 1 (SDF-1), is expressed as three alternatively spliced isoforms (α, β, and γ). Within the CNS, the expression patterns for CXCL12 is widespread and includes the cortex, olfactory bulb, hippocampus, cerebellum, meninges, and the endothelium of the BBB (β and γ isoforms). Further, expression of CXCR4, the receptor for CXCL12, has been detected in numerous cell types in the CNS including astrocytes, microglia, oligodendrocytes, neurons, and endothelial cells of the BBB (van der Meer et al., 2000; Stumm et al., 2002). Until recently it was believed that CXCL12 mediated its effects exclusively via interactions with CXCR4, however the receptor CXCR7/ACKR3 (Atypical chemokine receptor 3), formerly the orphan receptor RDC1, has now been shown to bind CXCL12 as well as CXCL11 (Burns et al., 2006). While CXCR7/ACKR3 (CXCR7) possesses homology with conserved domains of G-protein coupled receptors and is structurally similar to other CXC receptors, ligand binding does not initiate typical intracellular signaling pathways but instead results in β-arrestin recruitment and MAP kinase activation (Rajagopal et al., 2010; Odemis et al., 2012). One function of CXCR7 appears to be its ability to act as a scavenger receptor for both CXCL12 and CXCL11, mediating uptake and degradation of these ligands, and thus regulating extracellular chemokine concentrations (Boldajipour et al., 2008; Naumann et al., 2010). The ability of CXCR7 to act as a sink for CXCL12 may have implications for controlling chemokine gradients and directing hematopoetic cells, leukocytes and other cell subsets to peripheral lymphoid tissues as well as the CNS. CXCR7 expression within the CNS of rats was detected via *in situ* hybridization, with CXCR7 mRNA transcripts identified in the ventricular ependyma, the choroid plexus, neuronal and astroglial cells as well as cells of the vasculature (Schönemeier et al., 2008a,b). Interestingly, studies have identified the endothelium of the BBB as a source of constitutive expression of CXCL12 and CXCR4 as well as CXCR7, suggesting a role for this chemokine/receptor axis in regulating immune cell trafficking at the BBB during homeostasis.

These initial observations of CXCL12/CXCR4 expression by BBB endothelial cells were expanded by McCandless et al. who demonstrated the importance of CXCL12 expression and polarization at the BBB in the perivascular localization of infiltrating mononuclear cells (McCandless et al., 2006). These studies determined that CXCL12 protein is normally localized along the abluminal surface of endothelium within the CNS of mice and humans. During the autoimmune diseases experimental autoimmune encephalomyelitis (EAE) in mice and multiple sclerosis (MS) in humans, CXCL12 localization shifts toward a more luminal expression pattern, which is accompanied by increased parenchymal entry of CXCR4 positive mononuclear cells. Loss of CXCL12 polarity and luminal display of this chemokine was also associated with the detection of activated CXCR4 on leukocytes within the blood, suggesting that relocation of CXCL12 in this manner not only promotes the egress of leukocytes from perivascular spaces but also increases their capture and translocation across the BBB. These results suggest that abluminal expression of CXCL12 at the CNS vasculature during homeostatic conditions is a component of immune privilege essential for limiting extravasation of circulating leukocytes across endothelial barriers, while restricting immune cells to the perivascular space, limiting their access to parenchymal tissues (Figure 2).

### Chemokine Signaling at CNS Barriers in Health and Disease

#### The CXCL12, CXCR4, CXCR7 axis

More recently, the role of CXCR7 in regulating CXCL12 polarity at the BBB was examined by Cruz-Orengo et al. (2011). Consistent with earlier reports (Schönemeier et al., 2008a,b), results from this study found constitutive CXCR7 expression by the CNS vasculature. In addition, CXCR7 message was detected in primary cultures of murine brain microvascular endothelial cells (BMECs). During EAE, CXCR7 levels increased at postcapillary venules of spinal cord white matter, with concomitant loss of CXCL12 polarity at the BBB (Figure 2). Administration of an antagonist to the CXCR7 receptor prevented loss of abluminal CXCL12, limiting leukocyte entry at the BBB and the formation of parenchymal inflammatory lesions. *In vitro* experiments using murine BMECs determined that proinflammatory T cell cytokines, interleukin (IL)-1β and IL-17, increased expression of CXCL12 and CXCR7, respectively, with increased localization of CXCL12 with lysosomal markers. Further, these cytokines enhanced uptake of exogenous CXCL12, which was inhibited in a dose-dependent manner by antagonism of CXCR7, suggesting that under inflammatory conditions, CXCR7 facilitates internalization of CXCL12, leading to loss of polarity at the BBB seen in EAE as well as MS (Cruz-Orengo et al., 2011).

Taken together, these results suggest a critical role for CXCR7 in mediating CXCL12 abundance and localization during neuroinflammation, but importantly may offer clues to the role of this chemokine/receptor axis during homeostasis. In the absence of inflammation, CXCL12 expression is localized on the abluminal surface of the BBB, despite constitutive expression of CXCR7 at the CNS microvasculature. This perhaps indicates that CXCR7 normally functions to maintain and replenish basal CXCL12 through internalization and recycling to the cell surface, as has been demonstrated in cell lines engineered to express CXCR7 (Luker et al., 2010) and in studies of germ cell migration (Mahabaleshwar et al., 2012). At this time, it is unclear if CXCR7 plays a similar role in mediating extravasation of immune cells across the BBB, however the maintenance of CXCL12 polarity during homeostatic conditions indicates that this mechanism may be an essential function of CXCR7 in non-pathogenic states.

#### CCL2 and CCR2

CCL2 is also known as monocyte chemoattractant protein-1 (MCP-1), an inflammatory chemokine expressed by immune cells as well as other stromal cell types. In response to inflammatory cues or tissue injury, CCL2 is up-regulated to recruit CCR2^+^ monocytes, memory T cells, and DCs (Kolattukudy and Niu, 2012). Within the CNS, CCL2 is expressed by neurons, astrocytes, and microvascular endothelial cells of the BBB, and the role of CCL2 in recruiting monocytes and macrophages into the CNS under inflammatory conditions has been well characterized (Conductier et al., 2010; Réaux-Le Goazigo et al., 2013). Nevertheless, recent studies have also demonstrated a role for CCL2 under homeostatic conditions and suggest that the expression of both CCL2 and CCR2 is necessary for perivascular and meningeal macrophage recruitment and turnover in the brains of mice (Schilling et al., 2009). Additionally, Stowe et al. have shown that hypoxic preconditioning of mice with 8% oxygen for 4 h led to an up-regulation of CCL2 by neurons as well as cerebral endothelial cells that was associated with increased tolerance to subsequent cerebral ischemia. This study also found that hypoxic preconditioning and up-regulation of CCL2 at the cerebral microvasculature was not sufficient to increase monocyte trafficking across the BBB (Stowe et al., 2012). These results suggest that CCL2 levels on microvascular endothelial cells can be up-regulated in the absence of inflammation and may function to confer a neuroprotective phenotype at the BBB.

### Leukocyte Homing in Cerebrospinal Fluid

The intensity of immune responses in the CNS increases with proximity to the ventricles of the brain (Matyszak and Perry, 1996), and materials implanted within the subarachnoid space and meninges are likewise capable of eliciting a robust immune response. These observations suggest that the ventricular and subarachnoid CSF may function as sites of physiological immune surveillance. Consistent with this, cellular infiltrates that accumulate within the meningeal membranes during neuroinflammatory events have been observed to arrange in formations resembling secondary lymphoid structures (Howell et al., 2011), while infiltrates within the parenchyma do not exhibit the features of lymphoid neogenesis.

#### The CCL19, CCL21, and CCR7 axis

In peripheral lymphoid tissues, the chemokine CCL19 guides CCR7-expressing B cells, naïve T cells, and DCs into lymphoid tissue under physiological conditions (Bachmann et al., 2006). Along with the chemokine, CCL21, CCL19 is constitutively expressed in lymphoid tissues including the spleen, Peyer’s patches, and lymph nodes, where they regulate homing of leukocytes (e.g., CCR7^+^ naïve T cells and mature DCs) and facilitate antigen-specific interactions within subcompartments of secondary lymphoid tissue (e.g., T cell zones and high endothelial venules). Thus CCL19 and CCL21 serve to generate adaptive immune responses and are critical for developing and maintaining secondary lymphoid tissues in the periphery and have also been implicated in lymphoid neogenesis within the CNS.

Kivisäkk et al. have shown that CD4^+^ T cells are restimulated within the subarachnoid space by encounters with MHC II^+^ antigen presenting cells prior to the onset of inflammation in EAE, providing further support to the concept of the subarachnoid space and meninges as a site of routine immunological surveillance (Kivisäkk et al., 2003, 2009). Studies from this group have also characterized the phenotype of leukocytes in the CSF of patients without CNS inflammation and found that these cells predominantly are CD4^+^/CD45RA^−^/CD27^+^/CD69^+^, consistent with the profile of activated central memory T cells that also express high levels of CCR7 and L-selectin. Given that CCL21, a ligand for CCR7, has been detected at the choroid plexus epithelium (Kivisäkk et al., 2004), it is possible that this chemokine directs CCR7^+^ activated memory T cells to cross the blood-CSF barrier during homeostatic immune surveillance of the CNS (Figure 1). The expression of CCR7 by these memory T cells also suggests that this chemokine receptor is important in maintaining these lymphocytes within the CNS, perhaps via in interactions with CCL19 or CCL21 expressed at the brain vasculature during physiologic as well as neuroinflammatory conditions. Additionally, the expression of CCR7 by memory T cells within the CSF may facilitate homing back to peripheral lymphoid tissue, particularly the deep cervical lymph nodes via drainage of the CSF across the cribriform plate and nasal mucosa (Goldmann et al., 2006; Laman and Weller, 2013).

CCL19 mRNA transcripts are constitutively expressed on the endothelial cells of post-capillary venules in the brain and spinal cord under physiologic conditions, while expression of CCL21, another CCR7 ligand, is induced at post-capillary venules only during neuroinflammation (Alt et al., 2002; Krumbholz et al., 2007). CCL19 transcripts were also detected in normal human brain homogenates while expression levels were elevated in homogenates from active and inactive MS lesions. Kivisäkk et al. examined the expression of CCR7, CCL19, and CCL21 in brain autopsy material and CSF samples from MS patients. In contrast to previous observations in mice, this study reported a lack of CCL19 or CCL21 protein expression in endothelial or parenchymal cells of non-lesioned white matter or in active or chronic MS lesions, but did find strong CCL21 immunoreactivity within the choroid plexus epithelium (Kivisäkk et al., 2004). The expression of these lymphoid chemokines in the CNS implies that CCL19, and to a lesser extent CCL21, may signal circulating leukocytes that normally home to peripheral lymphoid tissues. Based on the constitutive expression of CCL19 within the CNS, it is possible that this lymphoid chemokine may also function in physiological immune surveillance of the CNS, perhaps at the level of the postcapillary venules of the BBB by recruiting and retaining T cells, as well as other cells known to express CCR7 (e.g., B cells; Figure 1).

#### CCL20 and CCR6

The receptor CCR6 is unique among chemokine receptors in that it binds a single chemokine ligand, CCL20. In the periphery, CCR6 regulates mucosal immunity via several mechanisms, including mediating the recruitment of DCs to epithelial barriers during inflammation and homing of helper T cells and DCs to the mucosal lymphoid tissue of the gut (Ito et al., 2011). Accordingly, CCL20 is constitutively expressed at epithelial barriers of the skin, lungs, gut and choroid plexus, typically at low levels under non-pathologic conditions. However, in response to proinflammatory cytokines, CCL20 levels can be substantially up-regulated. In addition to recruiting leukocytes to mucosal barriers, recent experiments have implicated CCL20/CCR6 in the trafficking of T cells to the CNS across the choroid plexus during immune surveillance as well as neuroinflammation (Figure 1). Reboldi et al. demonstrated that CCL20 was constitutively expressed by the choroid plexus epithelium in both mice and humans, but was not expressed by endothelial cells of the parenchymal microvessels. Mice lacking CCR6, which is expressed on IL-17-producing T cells (Th17 cells), were highly resistant to active induction of EAE by MOG immunization. Further, in these CCR6-deficient mice, CD45^+^ cells accumulated within the parenchyma of the choroid plexus, but failed to enter the CNS. Passive transfer of EAE into CCR6 knockout mice using MOG-specific WT T cells was able to rescue disease susceptibility and led to recruitment of T cells, including those lacking CCR6, into the CNS parenchyma. This finding suggests that the initial trigger for inflammation in this EAE model was due to a CCR6-dependent entry of Th17 cells into the uninflamed CNS via trafficking across the CCL20-expressing choroid plexus epithelium, leading to a second wave of infiltration of lymphocytes that does not rely on CCR6 (Reboldi et al., 2009). The results from this study led these authors to speculate that CCL20/CCR6 is critical for surveillance of the CNS via the CSF and subarachnoid spaces.

CCL20 and CCR6 may also play a role in the entry of autoreactive T cells during EAE at the dorsal blood vessels of the fifth lumbar vertebrae (L5) of the spinal cord via an IL-6-mediated mechanism. Arima et al. have demonstrated that CCL20 is normally expressed by vasculature at this site. IL-6 mediated upregulation of CCL20 was induced by stimulating the soleus muscle, which led to the infiltration of autoreactive T cells across the BBB in an adoptive transfer model of EAE. These experiments provided evidence that sensory stimulation can trigger chemokine-mediated accumulation of T cells within the CNS microvasculature at specific locations within the lumbar spinal cord. These observations led the authors to postulate that expression of CCL20 at the dorsal L5 blood vessels, even in the absence of pathogenic T cells, could represent a “gateway” by which leukocytes expressing CCR6 could cross the BBB and gain entry into the CNS parenchyma (Arima et al., 2012).

## Chemokines and Maintenance of the Adult CNS

### Progenitor and Endothelial Cell Function

At one time, loss of neurons was thought to be irreversible in the adult brain; we now know that generation of replacement cells is an ongoing process in rodents and humans (Kuhn et al., 1996; Eriksson et al., 1998). Adult neurogenesis occurs in localized “neurogenic niches” from precursors that reside adjacent to the lateral ventricles, in the subventricular zone (SVZ) and in the subgranular zone (SGZ) of the hippocampus (Gage, 2000; Doetsch and Scharff, 2001; Alvarez-Buylla and Lim, 2004; Zhao et al., 2008; Sanai et al., 2011). Stem cells require extracellular signals produced by the CNS milieu to regulate their ability to self-renew, proliferate, and differentiate (Sanai et al., 2011). Cues within these local microenvironments perpetuate new neurons and facilitate their integration into the existing brain circuitry (van Praag et al., 2002; Zhao et al., 2008). Neural progenitor cells (NPCs) give rise to adult neurons, astrocytes, and oligodendrocytes, and can be classified into several lineages. GFAP^+^ astrocyte-like type B cells line the lateral ventricle and extend processes into the SVZ plexus blood vessels (Mirzadeh et al., 2008; Shen et al., 2008; Tavazoie et al., 2008). During lineage progression, type B cells give rise to transit amplifying type C cells (Pastrana et al., 2009). Type C cells rapidly divide and give rise to type A neuroblasts, which proliferate as they migrate in chains along blood vessels (Shen et al., 2008; Tavazoie et al., 2008). The endothelial cells that make up the BBB vasculature serve as neurogenic “highways”, mediating progenitor cell trafficking and differentiation by providing external signage as guidance cues.

The CNS endothelium is essential for maintenance and homeostasis of the neural progenitor pool. In the adult, bone marrow-derived endothelial progenitor cells (EPCs) express CXCR4 and respond to CXCL12 as well as other cytokines to augment neovascularization for restoration of homeostasis following CNS injury (Zhang et al., 2002; Zheng et al., 2007; Yamaguchi et al., 2003). These epithelial cells directly or indirectly give rise to all the neurons, astrocytes, and oligodendrocytes in the adult brain (Deverman and Patterson, 2009). Not only does the endothelium give rise to CNS progenitors, the neurogenic niche is localized around the vasculature. Approximately 47% of dividing progenitor (type B cells) and 46% of transit amplifying (type C) cells are located within 5 microns of the endothelium (Shen et al., 2004, 2008). These progenitors directly contact the vessels of the SVZ in areas devoid of astrocyte end-feet and pericyte coverage, suggesting the vasculature endothelium, in particular, is an essential matrix and source of external cues for NPCs (Shen et al., 2008; Teng et al., 2008).

### The CXCL12/CXCR4/CXCR7 Axis and Neural Progenitor Cells

**Figure 3:**
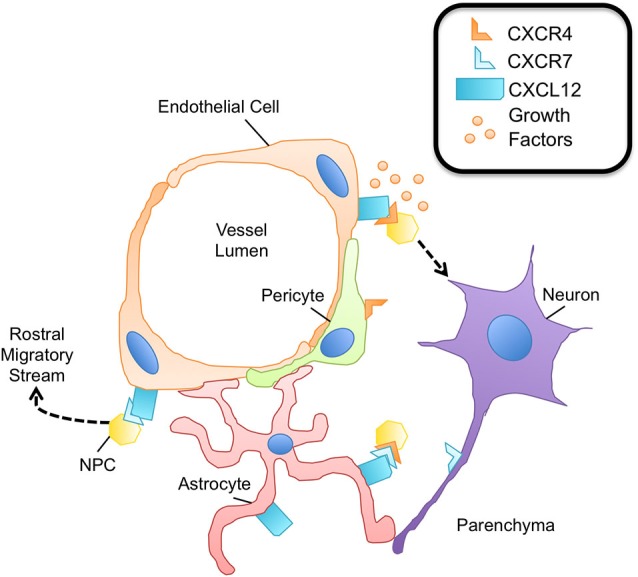
**The neurovascular unit promotes NPC survival.** Within the neurovascular unit, multiple cell types promote the survival and maintenance of NPCs via expression of the CXCL12/CXCR4/CXCR7 chemokine axis. NPCs express both CXCR4 and CXCR7, capable of heterodimerization. Binding of this receptor complex to CXCL12 leads to NPC survival via phosphorylation of ERK1/2. The binding of CXCL12 to CXCR4 on NPCs promotes adhesion molecule expression for enhanced attachment of NPCs to endothelial cells, a rich source of growth factors important for NPC survival and proliferation. CXCR4 activation on NPCs is also critical to sustain the pool of neural-committed progenitor cells within neurogenic niches. Pericytes are thought to express CXCR4; however the downstream effects of CXCR4 signaling in pericytes are largely unknown. CXCR7 expression is known to contribute to the migratory capacity of NPCs, particularly in the context of entering the rostral migratory stream and traveling to the olfactory bulb. Axons have also been shown to express CXCR7 during development as well as during neuroinflammation, although its function is largely not understood.

Chemokines expressed by the vasculature in the adult CNS are dynamically or constitutively regulated to provide migratory, proliferative, or differentiation cues to neurons and glia (Deverman and Patterson, 2009). Similar to hematopoietic progenitor cells in the bone marrow, in which CXCR4-CXCL12 signaling maintains the progenitor pool (Sugiyama et al., 2006), proliferative SVZ progenitor cells home to endothelial cells in the CNS in a CXCL12- and CXCR4-dependant manner under physiologic conditions. Kokovay et al. demonstrated that in early progenitor and transit amplifying cells, epidermal growth factor receptor and α6 integrin is up-regulated downstream of CXCL12 binding, enhancing the ability of activated NPCs to bind laminin on the CNS vasculature (Figure 3). Further, they showed that CXCL12 regulates the migration of neuroblasts from the SVZ (Kokovay et al., 2010), suggesting that endothelial CXCL12 can regulate progenitor cell occupancy of and departure from the vasculature niche of the adult SVZ. Similarly, in the SGZ, Schultheiss et al. have recently demonstrated that neuronal-committed progenitor cells express CXCR4 and that CXCR4 is phosphorylated in a CXCL12-dependent fashion (Figure 3). Further, deletion of CXCR4 in NPCs of adult mice resulted in reduced neurogenesis, specifically, a reduction in Sox2^+^ early progenitors, NeuroD^+^ neuronal-committed progenitors, and doublecortin^+^ immature neurons was observed (Schultheiß et al., 2013). Together, these studies suggest that CXCL12-mediated CXCR4 activation is required for maintenance of NPCs in neurogenic zones of the adult CNS.

Multiple pathways are known to regulate CXCR4 activation in neurogenesis. During development, chemokines position neural progenitors in the SGZ such that they are exposed to a range of neurogenic factors, including Wnt and Sonic hedgehog (Shh; Klein et al., 2001; Machold et al., 2003). These factors are critical for the maintenance of adult neurogenesis as Shh promotes proliferation of NPCs (Machold et al., 2003) and manipulation of the Wnt pathway nearly abolishes neurogenesis in the adult hippocampus (Lie et al., 2005). It is now known that CXCR4 is a downstream target of Wnt signaling, suggesting that Wnt induces CXCL12-mediated processes in NPCs via receptor regulation (Choe and Pleasure, 2012).

The alternate receptor for CXCL12, CXCR7, has also been shown to have a prominent role in adult neurogenesis. Zhu et al. demonstrated that both CXCR4 and CXCR7 are required for the survival of human NPCs. While CXCR4 is broadly expressed on the surface of human NPCs, CXCR7 was primarily localized in early endosomes, quickly trafficking to the plasma membrane to mediate CXCL12 endocytosis. Treatment of human NPCs with exogenous CXCL12, however, led to CXCR4/CXCR7 colocalization and downstream ERK1/2 signaling, which was shown to be essential for NPC survival (Zhu et al., 2012). In another study, CXCR7 expression regulated the migratory behavior of early neurons in the forebrain. CXCR7, but not CXCR4, was expressed by olfactory interneuron precursors, and down-regulation of CXCR7 impacted the ability of the precursors to integrate into the rostral migratory stream, the pathway to the olfactory bulbs (Tiveron et al., 2010). These studies suggest that CXCR7 has a prominent role in adult neurogenesis both in the context of and independent of CXCR4 signaling (Figure 3).

The CXCL12/CXCR4/CXCR7 axis has multiple roles in the homeostasis of adult neurogenesis. However, it is becoming increasingly clear that this axis may be up-regulated following injury including stroke (Hill et al., 2004; Wang et al., 2012), traumatic brain injury (Israelsson et al., 2008), or demyelination (Carbajal et al., 2010, 2011; Patel et al., 2010, 2012; Williams et al., 2014) to generate replacement cells and restore normal CNS function.

### CXCL12 and CXCL14 Regulate Hippocampal Neurogenesis

Following homology cloning, phylogenetic analysis revealed that CXCL14 is one of the oldest chemokines (Huising et al., 2004), yet its functions are relatively unknown. Considered a homeostatic chemokine, CXCL14 (BRAK; breast and kidney derived) is constitutively expressed in many regions of the brain (Huising et al., 2004) including the cortex, basal ganglia, septum, hippocampus, and hypothalamus (Banisadr et al., 2011; Yamamoto et al., 2011). It is thought to have an opposing role to CXCL12 (Banisadr et al., 2011; Tanegashima et al., 2013) due to its ability to bind a shared receptor, CXCR4 (Tanegashima et al., 2013). CXCL14 is highly expressed in many regions of the adult brain, including the hippocampus, and may regulate synaptic inputs to adult NPCs (Banisadr et al., 2011). The early development of NPCs within the SGZ is regulated by excitatory GABAergic synaptic inputs that promote synaptic maturity (Ge et al., 2007; Ma et al., 2009). These newborn neurons mature and are synaptically integrated into the dentate gyrus of the hippocampus (van Praag et al., 2002). GABAergic synapses on adult NPCs are sensitive to CXCL12 and CXCL14, which enhance (Bhattacharyya et al., 2008) and inhibit the effects of GABA (Banisadr et al., 2011), respectively. These findings suggest that CXCL12 and CXCL14 work to regulate hippocampal integrity in mature mammals. This is consistent with experiments using a CXCR4 antagonist in adult mice where blockade of CXCR4 signaling impaired recognition and memory (Parachikova and Cotman, 2007). Taken together, these studies suggest that CXCL12 and CXCL14 have an opposing role, regulating NPC responses to synaptic stimulation, and maintaining balance in homeostatic NPC turnover in the adult brain.

### CX3CL1 Maintains the Neurogenic Niche

CX_3_CL1 (fractalkine) is much longer than most chemokines (373 vs. ∼80 AAs) and also exists in two forms: a 95 kDa membrane-bound form with an N-terminal chemokine domain, a glycosylated mucin-like stalk, a hydrophobic transmembrane region and an intracellular C-terminal domain; and a 70 kDa soluble form that contains only the N-terminal chemokine domain. The soluble chemokine domain of CX_3_CL1, when cleaved, can act as a signaling molecule (Chapman et al., 2000), inducing chemotaxis in T cells and monocytes (Hermand et al., 2008), whereas its membrane-tethered mucin stalk can serve as a cell adhesion molecule, via binding of the CX_3_CL1 receptor, CX_3_CR1 (Haskell et al., 1999). Under physiologic conditions, CX_3_CL1 is highly expressed by a variety of neurons throughout the CNS (Hatori et al., 2002), with especially high levels in hippocampal neurons (Sheridan and Murphy, 2013). Neurons and microglia both express its receptor, CX_3_CR1 (Hatori et al., 2002), which regulates memory formation and synaptic plasticity via direct effects on glutamatergic synapses (Hoshiko et al., 2012; for an extensive review, see Sheridan and Murphy, 2013).

CX_3_CL1 has been shown to play a key role in maintaining adult neurogenesis via indirect mechanisms that modify the CNS microenvironment. CX_3_CL1 normally limits microglial activation and expression of proinflammatory cytokines including IL-1β, IL-6, and tumor necrosis factor (TNF)-α (Bachstetter et al., 2011; Rogers et al., 2011), which act directly on NPCs (Monje et al., 2003; Iosif et al., 2006; Koo and Duman, 2008). Thus, both genetic and antibody-based blockade of CX_3_CR1 signaling attenuates the inhibition of microglial activation and impacts hippocampal neurogenesis in adult animals. With age, there is an increase in activated microglia, which can promote an inflammatory milieu (Gemma et al., 2007) and contribute to age-related declines in neurogenesis (Rao et al., 2006; Ben Abdallah et al., 2010). Both exogenous CX_3_CL1 or IL-1R antagonist reverse this decline in aged animals. In addition, cleaved CX_3_CL1 acts as a sensor for neuronal stress, which stimulates microglia to phagocytose excitotoxic neurons (Noda et al., 2011). Given the many facets in which CX_3_CL1/CX_3_CR1 signaling works to inhibit inflammation and maintain a milieu skewed towards quiescence, it is probable that CX_3_CL1 contributes to the preservation of an optimal neurogenic niche for the development, proliferation, and integration of NPCs within the adult CNS.

Taken together, CX_3_CL1 plays an important role in maintenance of homeostasis in the adult CNS by mediating neuron-microglia interactions during physiologic conditions. Following CX_3_CR1 activation, microglia are known to remove excess neurons and support maturing synapses (Hoshiko et al., 2012; Cunningham et al., 2013; Lenz et al., 2013; Ueno et al., 2013), eliminate apoptotic neural progenitors during adult hippocampal neurogenesis (Sierra et al., 2014), and remodel neuronal circuitry during learning and memory processes (Schafer et al., 2012; Parkhurst et al., 2013). Unmanipulated adult mice deficient in CX_3_CR1 had a reduction in “synaptic multiplicity”, in which fewer boutons synapsed with more than one postsynaptic spine on a single dendrite. This resulted in reduced connectivity strength between regions of the hippocampus (Zhan et al., 2014), suggesting that CX_3_CL1 is required for adult hippocampal plasticity. It is clear that CX_3_CL1 is crucial in maintaining homeostasis in the adult CNS; however mechanisms downstream of membrane-bound CX_3_CL1- CX_3_CR1 binding, as pertains to normal physiology, remain to be fully elucidated.

## Concluding Remarks

While chemokines have historically been thought of as mediators of cell migration, recent evidence suggests that chemokines have the capacity to regulate a number of cellular functions critical to inflammatory processes as well as maintenance of homeostasis. Though the characterization of homeostatic chemokines stems from their roles in lymphoid tissues, many parallels can be drawn in the CNS, particularly in the context of the vasculature. Several chemokines contribute to immune cell trafficking and activation during immunosurveillance (CCL2, CCL19, CCL20, CCL21, CXCL12); regulation of neural progenitor cell migration, proliferation, differentiation, and integration (CXCL12, CXCL14); and maintenance of quiescence (CX_3_CL1), orchestrating the balance of homeostasis, while providing immune protection, under physiologic conditions in the CNS. While many functions of chemokines during homeostasis have been identified, there is still much to learn.

Chemokines are known to participate in the function of pericytes within the CNS during physiologic conditions, particularly at the level of the vasculature; however, the role of many of these chemokines is still largely unknown. Pericytes are a key component to the neurovascular unit within the CNS, contributing to endothelial cell tight junction stability and BBB formation (Balabanov and Dore-Duffy, 1998). Song et al. demonstrated that CXCL12 increases pericyte motility *in vitro* and in a tumor xenograft model *in vivo* (Song et al., 2009), suggesting that pericytes express CXCR4 that facilitates their recruitment to endothelial cells (Virgintino et al., 2013). Vascular pericytes have also been shown to respond to ligands of CXCR3, inducing chemotactic as well as mitogenic effects, stimulating proliferation (Bonacchi et al., 2001). Further, pericytes are known to be a source of chemokine ligands, including CCL3 and CCL4, both constitutively and in response to LPS (Kovac et al., 2011). Due to the spatial distribution of pericytes within the neurovascular unit (Figure 3), they are likely to have an important role with regard to maintenance of homeostasis via chemokine regulation. Elucidating the role of chemokines in pericyte function will aid in the understanding of these cells in the context of homeostasis and disease in the CNS.

CCL27 (cutaneous T cell-attracting chemokine, CTACK) is largely expressed by keratinocytes, binds to the receptor CCR10, and may serve as an important regulator of homeostatic immune surveillance. CCL27 has been implicated in inflammatory allergic reactions, primarily in homing memory T cells to the skin (Morales et al., 1999; Huang et al., 2008). Interestingly, Gunsolly et al. have characterized the expression of CCL27 in the cerebral cortex and limbic regions of the CNS in mice. During allergic inflammation induced by intranasal injection of ovalbumin, a variant of CCL27 was up-regulated in the olfactory bulb and was accompanied by infiltration of T cells (Gunsolly et al., 2010), suggesting that CCL27 also has a role in T cell recruitment in the CNS. This pathway may represent a point of access to the CNS tissue for leukocytes that bypasses the BBB, which is lacking at the nasal mucosa, cribriform plate, and perineural spaces of the olfactory bulb (Danielyan et al., 2009). Continued investigation of chemokines in the adult CNS will provide new insights into their functions during physiologic conditions and maintenance of CNS protection, and may identify targets for restoring homeostasis following CNS injury.

## Conflict of interest statement

The authors declare that the research was conducted in the absence of any commercial or financial relationships that could be construed as a potential conflict of interest.

## References

[B1] Alt C. Laschinger M. Engelhardt B. (2002). Functional expression of the lymphoid chemokines CCL19 (ELC) and CCL 21 (SLC) at the blood-brain barrier suggests their involvement in G-protein-dependent lymphocyte recruitment into the central nervous system during experimental autoimmune encephalomyelitis. Eur. J. Immunol. 32, 2133–2144 10.1002/1521-4141(200208)32:8<2133::aid-immu2133>3.0.co;2-w 12209625

[B2] Alvarez-Buylla A. Lim D. A. (2004). For the long run: maintaining germinal niches in the adult brain. Neuron 41, 683–686 10.1016/S0896-6273(04)00111-4 15003168

[B3] Arima Y. Harada M. Kamimura D. Park J. H. Kawano F. Yull F. E. (2012). Regional neural activation defines a gateway for autoreactive T cells to cross the blood-brain barrier. Cell 148, 447–457 10.1016/j.cell.2012.01.022 22304915

[B4] Bachmann M. F. Kopf M. Marsland B. J. (2006). Chemokines: more than just road signs. Nat. Rev. Immunol. 6, 159–164 10.1038/nri1776 16491140

[B5] Bachstetter A. D. Morganti J. M. Jernberg J. Schlunk A. Mitchell S. H. Brewster K. W. (2011). Fractalkine and CX 3 CR1 regulate hippocampal neurogenesis in adult and aged rats. Neurobiol. Aging 32, 2030–2044 10.1016/j.neurobiolaging.2009.11.022 20018408 PMC2889032

[B6] Balabanov R. Dore-Duffy P. (1998). Role of the CNS microvascular pericyte in the blood-brain barrier. J. Neurosci. Res. 53, 637–644 10.1002/(sici)1097-4547(19980915)53:6<637::aid-jnr1>3.0.co;2-6 9753191

[B7] Banisadr G. Bhattacharyya B. J. Belmadani A. Izen S. C. Ren D. Tran P. B. (2011). The chemokine BRAK/CXCL14 regulates synaptic transmission in the adult mouse dentate gyrus stem cell niche. J. Neurochem. 119, 1173–1182 10.1111/j.1471-4159.2011.07509.x 21955359 PMC3330702

[B8] Ben Abdallah N. M. Slomianka L. Vyssotski A. L. Lipp H. P. (2010). Early age-related changes in adult hippocampal neurogenesis in C57 mice. Neurobiol. Aging 31, 151–161 10.1016/j.neurobiolaging.2008.03.002 18455269

[B9] Bhattacharyya B. J. Banisadr G. Jung H. Ren D. Cronshaw D. G. Zou Y. (2008). The chemokine stromal cell-derived factor-1 regulates GABAergic inputs to neural progenitors in the postnatal dentate gyrus. J. Neurosci. 28, 6720–6730 10.1523/jneurosci.1677-08.2008 18579746 PMC2720755

[B10] Boldajipour B. Mahabaleshwar H. Kardash E. Reichman-Fried M. Blaser H. Minina S. (2008). Control of chemokine-guided cell migration by ligand sequestration. Cell 132, 463–473 10.1016/j.cell.2007.12.034 18267076

[B11] Bonacchi A. Romagnani P. Romanelli R. G. Efsen E. Annunziato F. Lasagni L. (2001). Signal transduction by the chemokine receptor CXCR3: activation of Ras/ERK, Src and phosphatidylinositol 3-kinase/Akt controls cell migration and proliferation in human vascular pericytes. J. Biol. Chem. 276, 9945–9954 10.1074/jbc.m010303200 11136732

[B12] Burns J. M. Summers B. C. Wang Y. Melikian A. Berahovich R. Miao Z. (2006). A novel chemokine receptor for SDF-1 and I-TAC involved in cell survival, cell adhesion and tumor development. J. Exp. Med. 203, 2201–2213 10.1084/jem.20052144 16940167 PMC2118398

[B13] Carbajal K. S. Miranda J. L. Tsukamoto M. R. Lane T. E. (2011). CXCR4 signaling regulates remyelination by endogenous oligodendrocyte progenitor cells in a viral model of demyelination. Glia 59, 1813–1821 10.1002/glia.21225 21830237 PMC5025299

[B14] Carbajal K. S. Schaumburg C. Strieter R. Kane J. Lane T. E. (2010). Migration of engrafted neural stem cells is mediated by CXCL12 signaling through CXCR4 in a viral model of multiple sclerosis. Proc. Natl. Acad. Sci. U S A 107, 11068–11073 10.1073/pnas.1006375107 20534452 PMC2890772

[B15] Chapman G. A. Moores K. Harrison D. Campbell C. A. Stewart B. R. Strijbos P. J. (2000). Fractalkine cleavage from neuronal membranes represents an acute event in the inflammatory response to excitotoxic brain damage. J. Neurosci. 20, 1–5, RC87. 10899174 10.1523/JNEUROSCI.20-15-j0004.2000 PMC6772533

[B16] Charles J. Di Domizio J. Salameire D. Bendriss-Vermare N. Aspord C. Muhammad R. (2010). Characterization of circulating dendritic cells in melanoma: role of CCR6 in plasmacytoid dendritic cell recruitment to the tumor. J. Invest. Dermatol. 130, 1646–1656 10.1038/jid.2010.24 20220766

[B17] Chinnery H. R. Ruitenberg M. J. Mcmenamin P. G. (2010). Novel characterization of monocyte-derived cell populations in the meninges and choroid plexus and their rates of replenishment in bone marrow chimeric mice. J. Neuropathol. Exp. Neurol. 69, 896–909 10.1097/nen.0b013e3181edbc1a 20720507

[B18] Choe Y. Pleasure S. J. (2012). Wnt signaling regulates intermediate precursor production in the postnatal dentate gyrus by regulating CXCR4 expression. Dev. Neurosci. 34, 502–514 10.1159/000345353 23257686 PMC7962862

[B19] Clarkson B. D. Héninger E. Harris M. G. Lee J. Sandor M. Fabry Z. (2012). Innate-adaptive crosstalk: how dendritic cells shape immune responses in the CNS. Adv. Exp. Med. Biol. 946, 309–333 10.1007/978-1-4614-0106-3_18 21948376 PMC3666851

[B20] Conductier G. Blondeau N. Guyon A. Nahon J. L. Rovère C. (2010). The role of monocyte chemoattractant protein MCP1/CCL2 in neuroinflammatory diseases. J. Neuroimmunol. 224, 93–100 10.1016/j.jneuroim.2010.05.010 20681057

[B21] Cruz-Orengo L. Holman D. W. Dorsey D. Zhou L. Zhang P. Wright M. (2011). CXCR7 influences leukocyte entry into the CNS parenchyma by controlling abluminal CXCL12 abundance during autoimmunity. J. Exp. Med. 208, 327–339 10.1084/jem.20102010 21300915 PMC3039853

[B22] Cunningham C. L. Martínez-Cerdeño V. Noctor S. C. (2013). Microglia regulate the number of neural precursor cells in the developing cerebral cortex. J. Neurosci. 33, 4216–4233 10.1523/jneurosci.3441-12.2013 23467340 PMC3711552

[B23] Danielyan L. Schäfer R. Von Ameln-Mayerhofer A. Buadze M. Geisler J. Klopfer T. (2009). Intranasal delivery of cells to the brain. Eur. J. Cell Biol. 88, 315–324 10.1016/j.ejcb.2009.02.001 19324456

[B24] Deverman B. E. Patterson P. H. (2009). Cytokines and CNS development. Neuron 64, 61–78 10.1016/j.neuron.2009.09.002 19840550

[B25] Doetsch F. Scharff C. (2001). Challenges for brain repair: insights from adult neurogenesis in birds and mammals. Brain Behav. Evol. 58, 306–322 10.1159/000057572 11978948

[B26] Eriksson P. S. Perfilieva E. Björk-Eriksson T. Alborn A. M. Nordborg C. Peterson D. A. (1998). Neurogenesis in the adult human hippocampus. Nat. Med. 4, 1313–1317 10.1038/3305 9809557

[B27] Fernández-Arenas E. Calleja E. Martinez-Martin N. Gharbi S. I. Navajas R. Garcia-Medel N. (2014). Beta-arrestin-1 mediates the TCR-triggered re-routing of distal receptors to the immunological synapse by a PKC-mediated mechanism. EMBO J. 33, 559–577 10.1002/embj.201386022 24502978 PMC3989651

[B28] Gage F. H. (2000). Mammalian neural stem cells. Science 287, 1433–1438 10.1126/science.287.5457.1433 10688783

[B29] Galea I. Bechmann I. Perry V. H. (2007). What is immune privilege (not)? Trends Immunol. 28, 12–18 10.1016/j.it.2006.11.004 17129764

[B30] Ge S. Pradhan D. A. Ming G. L. Song H. (2007). GABA sets the tempo for activity-dependent adult neurogenesis. Trends Neurosci. 30, 1–8 10.1016/j.tins.2006.11.001 17116335

[B31] Gemma C. Bachstetter A. D. Cole M. J. Fister M. Hudson C. Bickford P. C. (2007). Blockade of caspase-1 increases neurogenesis in the aged hippocampus. Eur. J. Neurosci. 26, 2795–2803 10.1111/j.1460-9568.2007.05875.x 18001276

[B32] Ginhoux F. Greter M. Leboeuf M. Nandi S. See P. Gokhan S. (2010). Fate mapping analysis reveals that adult microglia derive from primitive macrophages. Science 330, 841–845 10.1126/science.1194637 20966214 PMC3719181

[B33] Goldmann J. Kwidzinski E. Brandt C. Mahlo J. Richter D. Bechmann I. (2006). T cells traffic from brain to cervical lymph nodes via the cribroid plate and the nasal mucosa. J. Leukoc. Biol. 80, 797–801 10.1189/jlb.0306176 16885505

[B34] Gunsolly C. Nicholson J. D. Listwak S. J. Ledee D. Zelenka P. Verthelyi D. (2010). Expression and regulation in the brain of the chemokine CCL27 gene locus. J. Neuroimmunol. 225, 82–90 10.1016/j.jneuroim.2010.04.019 20605223 PMC2924910

[B35] Hamel D. J. Sielaff I. Proudfoot A. E. Handel T. M. (2009). Chapter 4. Interactions of chemokines with glycosaminoglycans. Methods Enzymol. 461, 71–102 10.1016/s0076-6879(09)05404-4 19480915

[B36] Harrison J. K. Jiang Y. Chen S. Xia Y. Maciejewski D. Mcnamara R. K. (1998). Role for neuronally derived fractalkine in mediating interactions between neurons and CX3CR1-expressing microglia. Proc. Natl. Acad. Sci. U S A 95, 10896–10901 10.1073/pnas.95.18.10896 9724801 PMC27992

[B37] Haskell C. A. Cleary M. D. Charo I. F. (1999). Molecular uncoupling of fractalkine-mediated cell adhesion and signal transduction. Rapid flow arrest of CX3CR1-expressing cells is independent of G-protein activation. J. Biol. Chem. 274, 10053–10058 10.1074/jbc.274.15.10053 10187784

[B38] Hatori K. Nagai A. Heisel R. Ryu J. K. Kim S. U. (2002). Fractalkine and fractalkine receptors in human neurons and glial cells. J. Neurosci. Res. 69, 418–426 10.1002/jnr.10304 12125082

[B39] Hermand P. Pincet F. Carvalho S. Ansanay H. Trinquet E. Daoudi M. (2008). Functional adhesiveness of the CX3CL1 chemokine requires its aggregation. Role of the transmembrane domain. J. Biol. Chem. 283, 30225–30234 10.1074/jbc.m802638200 18725411 PMC2662081

[B40] Hill W. D. Hess D. C. Martin-Studdard A. Carothers J. J. Zheng J. Hale D. (2004). SDF-1 (CXCL12) is upregulated in the ischemic penumbra following stroke: association with bone marrow cell homing to injury. J. Neuropathol. Exp. Neurol. 63, 84–96 14748564 10.1093/jnen/63.1.84

[B41] Hoshiko M. Arnoux I. Avignone E. Yamamoto N. Audinat E. (2012). Deficiency of the microglial receptor CX3CR1 impairs postnatal functional development of thalamocortical synapses in the barrel cortex. J. Neurosci. 32, 15106–15111 10.1523/jneurosci.1167-12.2012 23100431 PMC6704837

[B42] Howell O. W. Reeves C. A. Nicholas R. Carassiti D. Radotra B. Gentleman S. M. (2011). Meningeal inflammation is widespread and linked to cortical pathology in multiple sclerosis. Brain 134(Pt. 9), 2755–2771 10.1093/brain/awr182 21840891

[B43] Huang V. Lonsdorf A. S. Fang L. Kakinuma T. Lee V. C. Cha E. (2008). Cutting edge: rapid accumulation of epidermal CCL27 in skin-draining lymph nodes following topical application of a contact sensitizer recruits CCR10-expressing T cells. J. Immunol. 180, 6462–6466 10.4049/jimmunol.180.10.6462 18453562 PMC6980374

[B44] Huising M. O. Van Der Meulen T. Flik G. Verburg-Van Kemenade B. M. (2004). Three novel carp CXC chemokines are expressed early in ontogeny and at nonimmune sites. Eur. J. Biochem. 271, 4094–4106 10.1111/j.1432-1033.2004.04347.x 15479238

[B45] Iosif R. E. Ekdahl C. T. Ahlenius H. Pronk C. J. Bonde S. Kokaia Z. (2006). Tumor necrosis factor receptor 1 is a negative regulator of progenitor proliferation in adult hippocampal neurogenesis. J. Neurosci. 26, 9703–9712 10.1523/jneurosci.2723-06.2006 16988041 PMC6674454

[B46] Israelsson C. Bengtsson H. Kylberg A. Kullander K. Lewen A. Hillered L. (2008). Distinct cellular patterns of upregulated chemokine expression supporting a prominent inflammatory role in traumatic brain injury. J. Neurotrauma 25, 959–974 10.1089/neu.2008.0562 18665806

[B47] Ito T. Carson W. F. T. Cavassani K. A. Connett J. M. Kunkel S. L. (2011). CCR6 as a mediator of immunity in the lung and gut. Exp. Cell Res. 317, 613–619 10.1016/j.yexcr.2010.12.018 21376174 PMC3063449

[B48] Johnson Z. Proudfoot A. E. Handel T. M. (2005). Interaction of chemokines and glycosaminoglycans: a new twist in the regulation of chemokine function with opportunities for therapeutic intervention. Cytokine Growth Factor Rev. 16, 625–636 10.1016/j.cytogfr.2005.04.006 15990353

[B49] Kierdorf K. Erny D. Goldmann T. Sander V. Schulz C. Perdiguero E. G. (2013). Microglia emerge from erythromyeloid precursors via Pu.1- and Irf8-dependent pathways. Nat. Neurosci. 16, 273–280 10.1038/nn.3318 23334579

[B50] Kivisäkk P. Imitola J. Rasmussen S. Elyaman W. Zhu B. Ransohoff R. M. (2009). Localizing central nervous system immune surveillance: meningeal antigen-presenting cells activate T cells during experimental autoimmune encephalomyelitis. Ann. Neurol. 65, 457–469 10.1002/ana.21379 18496841 PMC3305810

[B51] Kivisäkk P. Mahad D. J. Callahan M. K. Sikora K. Trebst C. Tucky B. (2004). Expression of CCR7 in multiple sclerosis: implications for CNS immunity. Ann. Neurol. 55, 627–638 10.1002/ana.20049 15122702

[B52] Kivisäkk P. Mahad D. J. Callahan M. K. Trebst C. Tucky B. Wei T. (2003). Human cerebrospinal fluid central memory CD4+ T cells: evidence for trafficking through choroid plexus and meninges via P-selectin. Proc. Natl. Acad. Sci. U S A 100, 8389–8394 10.1073/pnas.1433000100 12829791 PMC166239

[B53] Klein R. S. Rubin J. B. Gibson H. D. Dehaan E. N. Alvarez-Hernandez X. Segal R. A. (2001). SDF-1 alpha induces chemotaxis and enhances sonic hedgehog-induced proliferation of cerebellar granule cells. Development 128, 1971–1981 11493520 10.1242/dev.128.11.1971

[B54] Kokovay E. Goderie S. Wang Y. Lotz S. Lin G. Sun Y. (2010). Adult SVZ lineage cells home to and leave the vascular niche via differential responses to SDF1/CXCR4 signaling. Cell Stem Cell 7, 163–173 10.1016/j.stem.2010.05.019 20682445 PMC2916873

[B55] Kolattukudy P. E. Niu J. (2012). Inflammation, endoplasmic reticulum stress, autophagy and the monocyte chemoattractant protein-1/CCR2 pathway. Circ. Res. 110, 174–189 10.1161/CIRCRESAHA.111.243212 22223213 PMC3265021

[B56] Koo J. W. Duman R. S. (2008). IL-1beta is an essential mediator of the antineurogenic and anhedonic effects of stress. Proc. Natl. Acad. Sci. U S A 105, 751–756 10.1073/pnas.0708092105 18178625 PMC2206608

[B57] Kovac A. Erickson M. A. Banks W. A. (2011). Brain microvascular pericytes are immunoactive in culture: cytokine, chemokine, nitric oxide and LRP-1 expression in response to lipopolysaccharide. J. Neuroinflammation 8:139 10.1186/1742-2094-8-139 21995440 PMC3207972

[B58] Kremer K. N. Clift I. C. Miamen A. G. Bamidele A. O. Qian N. X. Humphreys T. D. (2011). Stromal cell-derived factor-1 signaling via the CXCR4-TCR heterodimer requires phospholipase C-beta3 and phospholipase C-gamma1 for distinct cellular responses. J. Immunol. 187, 1440–1447 10.4049/jimmunol.1100820 21705626 PMC3140596

[B59] Krumbholz M. Theil D. Steinmeyer F. Cepok S. Hemmer B. Hofbauer M. (2007). CCL19 is constitutively expressed in the CNS, up-regulated in neuroinflammation, active and also inactive multiple sclerosis lesions. J. Neuroimmunol. 190, 72–79 10.1016/j.jneuroim.2007.07.024 17825430

[B60] Kuhn H. G. Dickinson-Anson H. Gage F. H. (1996). Neurogenesis in the dentate gyrus of the adult rat: age-related decrease of neuronal progenitor proliferation. J. Neurosci. 16, 2027–2033 8604047 10.1523/JNEUROSCI.16-06-02027.1996 PMC6578509

[B61] Laman J. D. Weller R. O. (2013). Drainage of cells and soluble antigen from the CNS to regional lymph nodes. J. Neuroimmune Pharmacol. 8, 840–856 10.1007/s11481-013-9470-8 23695293 PMC7088878

[B62] Lenz K. M. Nugent B. M. Haliyur R. Mccarthy M. M. (2013). Microglia are essential to masculinization of brain and behavior. J. Neurosci. 33, 2761–2772 10.1523/JNEUROSCI.1268-12.2013 23407936 PMC3727162

[B63] Lie D. C. Colamarino S. A. Song H. J. Desire L. Mira H. Consiglio A. (2005). Wnt signalling regulates adult hippocampal neurogenesis. Nature 437, 1370–1375 10.1038/nature04108 16251967

[B64] Luker K. E. Steele J. M. Mihalko L. A. Ray P. Luker G. D. (2010). Constitutive and chemokine-dependent internalization and recycling of CXCR7 in breast cancer cells to degrade chemokine ligands. Oncogene 29, 4599–4610 10.1038/onc.2010.212 20531309 PMC3164491

[B65] Ma S. Olucha-Bordonau F. E. Hossain M. A. Lin F. Kuei C. Liu C. (2009). Modulation of hippocampal theta oscillations and spatial memory by relaxin-3 neurons of the nucleus incertus. Learn. Mem. 16, 730–742 10.1101/lm.1438109 19880588

[B66] Machold R. Hayashi S. Rutlin M. Muzumdar M. D. Nery S. Corbin J. G. (2003). Sonic hedgehog is required for progenitor cell maintenance in telencephalic stem cell niches. Neuron 39, 937–950 10.1016/s0896-6273(03)00593-2 12971894

[B67] Mahabaleshwar H. Tarbashevich K. Nowak M. Brand M. Raz E. (2012). beta-arrestin control of late endosomal sorting facilitates decoy receptor function and chemokine gradient formation. Development 139, 2897–2902 10.1242/dev.080408 22791893

[B68] Matyszak M. K. Perry V. H. (1996). The potential role of dendritic cells in immune-mediated inflammatory diseases in the central nervous system. Neuroscience 74, 599–608 10.1016/0306-4522(96)00160-1 8865208

[B69] McCandless E. E. Wang Q. Woerner B. M. Harper J. M. Klein R. S. (2006). CXCL12 limits inflammation by localizing mononuclear infiltrates to the perivascular space during experimental autoimmune encephalomyelitis. J. Immunol. 177, 8053–8064 10.4049/jimmunol.177.11.8053 17114479

[B70] McMenamin P. G. (1999). Distribution and phenotype of dendritic cells and resident tissue macrophages in the dura mater, leptomeninges and choroid plexus of the rat brain as demonstrated in wholemount preparations. J. Comp. Neurol. 405, 553–562 10.1002/(sici)1096-9861(19990322)405:4<553::aid-cne8>3.3.co;2-y 10098945

[B71] Mirzadeh Z. Merkle F. T. Soriano-Navarro M. Garcia-Verdugo J. M. Alvarez-Buylla A. (2008). Neural stem cells confer unique pinwheel architecture to the ventricular surface in neurogenic regions of the adult brain. Cell Stem Cell 3, 265–278 10.1016/j.stem.2008.07.004 18786414 PMC2613692

[B72] Monje M. L. Toda H. Palmer T. D. (2003). Inflammatory blockade restores adult hippocampal neurogenesis. Science 302, 1760–1765 10.1126/science.1088417 14615545

[B73] Morales J. Homey B. Vicari A. P. Hudak S. Oldham E. Hedrick J. (1999). CTACK, a skin-associated chemokine that preferentially attracts skin-homing memory T cells. Proc. Natl. Acad. Sci. U S A 96, 14470–14475 10.1073/pnas.96.25.14470 10588729 PMC24460

[B74] Naumann U. Cameroni E. Pruenster M. Mahabaleshwar H. Raz E. Zerwes H. G. (2010). CXCR7 functions as a scavenger for CXCL12 and CXCL11. PLoS One 5:e9175 10.1371/journal.pone.0009175 20161793 PMC2820091

[B75] Neumann H. Wekerle H. (2013). Brain microglia: watchdogs with pedigree. Nat. Neurosci. 16, 253–255 10.1038/nn.3338 23434975

[B76] Nimmerjahn A. Kirchhoff F. Helmchen F. (2005). Resting microglial cells are highly dynamic surveillants of brain parenchyma in vivo. Science 308, 1314–1318 10.1126/science.1110647 15831717

[B77] Noda M. Doi Y. Liang J. Kawanokuchi J. Sonobe Y. Takeuchi H. (2011). Fractalkine attenuates excito-neurotoxicity via microglial clearance of damaged neurons and antioxidant enzyme heme oxygenase-1 expression. J. Biol. Chem. 286, 2308–2319 10.1074/jbc.m110.169839 21071446 PMC3023525

[B78] Odemis V. Lipfert J. Kraft R. Hajek P. Abraham G. Hattermann K. (2012). The presumed atypical chemokine receptor CXCR7 signals through G(i/o) proteins in primary rodent astrocytes and human glioma cells. Glia 60, 372–381 10.1002/glia.22271 22083878

[B79] Parachikova A. Cotman C. W. (2007). Reduced CXCL12/CXCR4 results in impaired learning and is downregulated in a mouse model of Alzheimer disease. Neurobiol. Dis. 28, 143–153 10.1016/j.nbd.2007.07.001 17764962 PMC2198928

[B80] Parish C. R. (2006). The role of heparan sulphate in inflammation. Nat. Rev. Immunol. 6, 633–643 10.1038/nri1918 16917509

[B81] Parkhurst C. N. Yang G. Ninan I. Savas J. N. Yates J. R. 3rd Lafaille J. J. (2013). Microglia promote learning-dependent synapse formation through brain-derived neurotrophic factor. Cell 155, 1596–1609 10.1016/j.cell.2013.11.030 24360280 PMC4033691

[B82] Pastrana E. Cheng L. C. Doetsch F. (2009). Simultaneous prospective purification of adult subventricular zone neural stem cells and their progeny. Proc. Natl. Acad. Sci. U S A 106, 6387–6392 10.1073/pnas.0810407106 19332781 PMC2669396

[B83] Patel J. R. Mccandless E. E. Dorsey D. Klein R. S. (2010). CXCR4 promotes differentiation of oligodendrocyte progenitors and remyelination. Proc. Natl. Acad. Sci. U S A 107, 11062–11067 10.1073/pnas.1006301107 20534485 PMC2890706

[B84] Patel J. R. Williams J. L. Muccigrosso M. M. Liu L. Sun T. Rubin J. B. (2012). Astrocyte TNFR2 is required for CXCL12-mediated regulation of oligodendrocyte progenitor proliferation and differentiation within the adult CNS. Acta Neuropathol. 124, 847–860 10.1007/s00401-012-1034-0 22933014 PMC3508279

[B85] Prodinger C. Bunse J. Kruger M. Schiefenhovel F. Brandt C. Laman J. D. (2011). CD11c-expressing cells reside in the juxtavascular parenchyma and extend processes into the glia limitans of the mouse nervous system. Acta Neuropathol. 121, 445–458 10.1007/s00401-010-0774-y 21076838

[B86] Rajagopal S. Kim J. Ahn S. Craig S. Lam C. M. Gerard N. P. (2010). Beta-arrestin- but not G protein-mediated signaling by the “decoy” receptor CXCR7. Proc. Natl. Acad. Sci. U S A 107, 628–632 10.1073/pnas.0912852107 20018651 PMC2818968

[B87] Rao M. S. Hattiangady B. Shetty A. K. (2006). The window and mechanisms of major age-related decline in the production of new neurons within the dentate gyrus of the hippocampus. Aging Cell 5, 545–558 10.1111/j.1474-9726.2006.00243.x 17129216

[B88] Réaux-Le Goazigo A. Van Steenwinckel J. Rostene W. Melik Parsadaniantz S. (2013). Current status of chemokines in the adult CNS. Prog. Neurobiol. 104, 67–92 10.1016/j.pneurobio.2013.02.001 23454481

[B89] Reboldi A. Coisne C. Baumjohann D. Benvenuto F. Bottinelli D. Lira S. (2009). C-C chemokine receptor 6-regulated entry of TH-17 cells into the CNS through the choroid plexus is required for the initiation of EAE. Nat. Immunol. 10, 514–523 10.1038/ni.1716 19305396

[B90] Rogers J. T. Morganti J. M. Bachstetter A. D. Hudson C. E. Peters M. M. Grimmig B. A. (2011). CX3CR1 deficiency leads to impairment of hippocampal cognitive function and synaptic plasticity. J. Neurosci. 31, 16241–16250 10.1523/JNEUROSCI.3667-11.2011 22072675 PMC3236509

[B91] Sallusto F. Schaerli P. Loetscher P. Schaniel C. Lenig D. Mackay C. R. (1998). Rapid and coordinated switch in chemokine receptor expression during dendritic cell maturation. Eur. J. Immunol. 28, 2760–2769 10.1002/(sici)1521-4141(199809)28:09<2760::aid-immu2760>3.0.co;2-n 9754563

[B92] Sanai N. Nguyen T. Ihrie R. A. Mirzadeh Z. Tsai H. H. Wong M. (2011). Corridors of migrating neurons in the human brain and their decline during infancy. Nature 478, 382–386 10.1038/nature10487 21964341 PMC3197903

[B93] Schafer D. P. Lehrman E. K. Kautzman A. G. Koyama R. Mardinly A. R. Yamasaki R. (2012). Microglia sculpt postnatal neural circuits in an activity and complement-dependent manner. Neuron 74, 691–705 10.1016/j.neuron.2012.03.026 22632727 PMC3528177

[B94] Schilling M. Strecker J. K. Ringelstein E. B. Kiefer R. Schabitz W. R. (2009). Turn-over of meningeal and perivascular macrophages in the brain of MCP-1-, CCR-2- or double knockout mice. Exp. Neurol. 219, 583–585 10.1016/j.expneurol.2009.07.003 19615366

[B95] Schneider O. D. Weiss A. A. Miller W. E. (2009). Pertussis toxin signals through the TCR to initiate cross-desensitization of the chemokine receptor CXCR4. J. Immunol. 182, 5730–5739 10.4049/jimmunol.0803114 19380820 PMC2766007

[B96] Schönemeier B. Kolodziej A. Schulz S. Jacobs S. Hoellt V. Stumm R. (2008a). Regional and cellular localization of the CXCl12/SDF-1 chemokine receptor CXCR7 in the developing and adult rat brain. J. Comp. Neurol. 510, 207–220 10.1002/cne.21780 18615560

[B97] Schönemeier B. Schulz S. Hoellt V. Stumm R. (2008b). Enhanced expression of the CXCl12/SDF-1 chemokine receptor CXCR7 after cerebral ischemia in the rat brain. J. Neuroimmunol. 198, 39–45 10.1016/j.jneuroim.2008.04.010 18513805

[B98] Schultheiß C. Abe P. Hoffmann F. Mueller W. Kreuder A. E. Schutz D. (2013). CXCR4 prevents dispersion of granule neuron precursors in the adult dentate gyrus. Hippocampus 23, 1345–1358 10.1002/hipo.22180 23929505

[B99] Schulz M. Engelhardt B. (2005). The circumventricular organs participate in the immunopathogenesis of experimental autoimmune encephalomyelitis. Cerebrospinal Fluid Res. 2:8 10.5772/29792 16197544 PMC1262737

[B100] Serot J. M. Bene M. C. Foliguet B. Faure G. C. (2000). Monocyte-derived IL-10-secreting dendritic cells in choroid plexus epithelium. J. Neuroimmunol. 105, 115–119 10.1016/s0165-5728(99)00240-4 10742552

[B101] Serrats J. Schiltz J. C. Garcia-Bueno B. Van Rooijen N. Reyes T. M. Sawchenko P. E. (2010). Dual roles for perivascular macrophages in immune-to-brain signaling. Neuron 65, 94–106 10.1016/j.neuron.2009.11.032 20152116 PMC2873837

[B102] Shen Q. Goderie S. K. Jin L. Karanth N. Sun Y. Abramova N. (2004). Endothelial cells stimulate self-renewal and expand neurogenesis of neural stem cells. Science 304, 1338–1340 10.1126/science.1095505 15060285

[B103] Shen Q. Wang Y. Kokovay E. Lin G. Chuang S. M. Goderie S. K. (2008). Adult SVZ stem cells lie in a vascular niche: a quantitative analysis of niche cell-cell interactions. Cell Stem Cell 3, 289–300 10.1016/j.stem.2008.07.026 18786416 PMC2747473

[B104] Sheridan G. K. Murphy K. J. (2013). Neuron-glia crosstalk in health and disease: fractalkine and CX3CR1 take centre stage. Open Biol. 3:130181 10.1098/rsob.130181 24352739 PMC3877844

[B105] Sierra A. Beccari S. Diaz-Aparicio I. Encinas J. M. Comeau S. Tremblay M. E. (2014). Surveillance, phagocytosis and inflammation: how never-resting microglia influence adult hippocampal neurogenesis. Neural Plast. 2014:610343 10.1155/2014/610343 24772353 PMC3977558

[B106] Smith X. Schneider H. Kohler K. Liu H. Lu Y. Rudd C. E. (2013). The chemokine CXCL12 generates costimulatory signals in T cells to enhance phosphorylation and clustering of the adaptor protein SLP-76. Sci. Signal. 6:ra65 10.1126/scisignal.2004018 23901140

[B107] Song N. Huang Y. Shi H. Yuan S. Ding Y. Song X. (2009). Overexpression of platelet-derived growth factor-BB increases tumor pericyte content via stromal-derived factor-1alpha/CXCR4 axis. Cancer Res. 69, 6057–6064 10.1158/0008-5472.CAN-08-2007 19584297

[B108] Stowe A. M. Wacker B. K. Cravens P. D. Perfater J. L. Li M. K. Hu R. (2012). CCL2 upregulation triggers hypoxic preconditioning-induced protection from stroke. J. Neuroinflammation 9:33 10.1186/1742-2094-9-33 22340958 PMC3298779

[B109] Strazielle N. Ghersi-Egea J. F. (2000). Choroid plexus in the central nervous system: biology and physiopathology. J. Neuropathol. Exp. Neurol. 59, 561–574 10901227 10.1093/jnen/59.7.561

[B110] Stumm R. K. Rummel J. Junker V. Culmsee C. Pfeiffer M. Krieglstein J. (2002). A dual role for the SDF-1/CXCR4 chemokine receptor system in adult brain: isoform-selective regulation of SDF-1 expression modulates CXCR4-dependent neuronal plasticity and cerebral leukocyte recruitment after focal ischemia. J. Neurosci. 22, 5865–5878 12122049 10.1523/JNEUROSCI.22-14-05865.2002 PMC6757949

[B111] Sugiyama T. Kohara H. Noda M. Nagasawa T. (2006). Maintenance of the hematopoietic stem cell pool by CXCL12-CXCR4 chemokine signaling in bone marrow stromal cell niches. Immunity 25, 977–988 10.1016/j.immuni.2006.10.016 17174120

[B112] Tanegashima K. Suzuki K. Nakayama Y. Tsuji K. Shigenaga A. Otaka A. (2013). CXCL14 is a natural inhibitor of the CXCL12-CXCR4 signaling axis. FEBS Lett. 587, 1731–1735 10.1016/j.febslet.2013.04.046 23669361

[B113] Tavazoie M. Van Der Veken L. Silva-Vargas V. Louissaint M. Colonna L. Zaidi B. (2008). A specialized vascular niche for adult neural stem cells. Cell Stem Cell 3, 279–288 10.1016/j.stem.2008.07.025 18786415 PMC6864413

[B114] Teng H. Zhang Z. G. Wang L. Zhang R. L. Zhang L. Morris D. (2008). Coupling of angiogenesis and neurogenesis in cultured endothelial cells and neural progenitor cells after stroke. J. Cereb. Blood Flow Metab. 28, 764–771 10.1038/sj.jcbfm.9600573 17971789 PMC2744583

[B115] Tiveron M. C. Boutin C. Daou P. Moepps B. Cremer H. (2010). Expression and function of CXCR7 in the mouse forebrain. J. Neuroimmunol. 224, 72–79 10.1016/j.jneuroim.2010.05.011 20681075

[B116] Ueno M. Fujita Y. Tanaka T. Nakamura Y. Kikuta J. Ishii M. (2013). Layer V cortical neurons require microglial support for survival during postnatal development. Nat. Neurosci. 16, 543–551 10.1038/nn.3358 23525041

[B117] van der Meer P. Ulrich A. M. Gonzalez-Scarano F. Lavi E. (2000). Immunohistochemical analysis of CCR2, CCR3, CCR5 and CXCR4 in the human brain: potential mechanisms for HIV dementia. Exp. Mol. Pathol. 69, 192–201 10.1006/exmp.2000.2336 11115360

[B118] van Praag H. Schinder A. F. Christie B. R. Toni N. Palmer T. D. Gage F. H. (2002). Functional neurogenesis in the adult hippocampus. Nature 415, 1030–1034 10.1038/4151030a 11875571 PMC9284568

[B119] Virgintino D. Errede M. Rizzi M. Girolamo F. Strippoli M. Walchli T. (2013). The CXCL12/CXCR4/CXCR7 ligand-receptor system regulates neuro-glio-vascular interactions and vessel growth during human brain development. J. Inherit. Metab. Dis. 36, 455–466 10.1007/s10545-012-9574-y 23344887

[B120] Wang Y. Huang J. Li Y. Yang G. Y. (2012). Roles of chemokine CXCL12 and its receptors in ischemic stroke. Curr. Drug Targets 13, 166–172 10.2174/138945012799201603 22204316

[B121] Williams J. L. Patel J. R. Daniels B. P. Klein R. S. (2014). Targeting CXCR7/ACKR3 as a therapeutic strategy to promote remyelination in the adult central nervous system. J. Exp. Med. 211, 791–799 10.1084/jem.20131224 24733828 PMC4010893

[B122] Wilson E. H. Weninger W. Hunter C. A. (2010). Trafficking of immune cells in the central nervous system. J. Clin. Invest. 120, 1368–1379 10.1172/JCI41911 20440079 PMC2860945

[B123] Yamaguchi J. Kusano K. F. Masuo O. Kawamoto A. Silver M. Murasawa S. (2003). Stromal cell-derived factor-1 effects on ex vivo expanded endothelial progenitor cell recruitment for ischemic neovascularization. Circulation 107, 1322–1328 10.1161/01.cir.0000055313.77510.22 12628955

[B124] Yamamoto T. Yamashita A. Yamada K. Hata R. (2011). Immunohistochemical localization of chemokine CXCL14 in rat hypothalamic neurons. Neurosci. Lett. 487, 335–340 10.1016/j.neulet.2010.10.051 20974226

[B125] Zachariae C. O. (1993). Chemotactic cytokines and inflammation. Biological properties of the lymphocyte and monocyte chemotactic factors ELCF, MCAF and IL-8. Acta Derm. Venereol. Suppl. (Stockh) 181, 1–37 7901957

[B126] Zhan Y. Paolicelli R. C. Sforazzini F. Weinhard L. Bolasco G. Pagani F. (2014). Deficient neuron-microglia signaling results in impaired functional brain connectivity and social behavior. Nat. Neurosci. 17, 400–406 10.1038/nn.3641 24487234

[B127] Zhang Z. G. Zhang L. Jiang Q. Chopp M. (2002). Bone marrow-derived endothelial progenitor cells participate in cerebral neovascularization after focal cerebral ischemia in the adult mouse. Circ. Res. 90, 284–288 10.1161/hh0302.104460 11861416

[B128] Zhao C. Deng W. Gage F. H. (2008). Mechanisms and functional implications of adult neurogenesis. Cell 132, 645–660 10.1016/j.cell.2008.01.033 18295581

[B129] Zheng H. Fu G. Dai T. Huang H. (2007). Migration of endothelial progenitor cells mediated by stromal cell-derived factor-1alpha/CXCR4 via PI3K/Akt/eNOS signal transduction pathway. J. Cardiovasc. Pharmacol. 50, 274–280 10.1097/fjc.0b013e318093ec8f 17878755

[B130] Zhu B. Xu D. Deng X. Chen Q. Huang Y. Peng H. (2012). CXCL12 enhances human neural progenitor cell survival through a CXCR7- and CXCR4-mediated endocytotic signaling pathway. Stem Cells 30, 2571–2583 10.1002/stem.1239 22987307 PMC3969741

